# The p53/Adipose-Tissue/Cancer Nexus

**DOI:** 10.3389/fendo.2018.00457

**Published:** 2018-08-14

**Authors:** Kevin Zwezdaryk, Deborah Sullivan, Zubaida Saifudeen

**Affiliations:** ^1^Department of Microbiology and Immunology, Tulane University School of Medicine, New Orleans, LA, United States; ^2^Department of Pediatrics, Section of Nephrology, Tulane University School of Medicine, New Orleans, LA, United States

**Keywords:** p53, adipokines, obesity, cancer, white adipose tissue, metabolism

## Abstract

Obesity and the resultant metabolic complications have been associated with an increased risk of cancer. In addition to the systemic metabolic disturbances in obesity that are associated with cancer initiation and progression, the presence of adipose tissue in the tumor microenvironment (TME) contributes significantly to malignancy through direct cell-cell interaction or paracrine signaling. This chronic inflammatory state can be maintained by p53-associated mechanisms. Increased p53 levels that are observed in obesity exacerbate the release of inflammatory cytokines that fuel cancer initiation and progression. Dysregulated adipose tissue signaling from the TME can reprogram tumor cell metabolism. The links between p53, cellular metabolism and adipose tissue dysfunction and how they relate to cancer, will be presented in this review.

## Introduction

Cancers associated with obesity are estimated to account for up to 40% of all cancers diagnosed in the US (Centers for Disease Control and Prevention, CDC). Per CDC reports, the incidence of non-obesity related cancers showed a decline from 2005 to 2014, while the rates of obesity-related cancers increased (1). Causes of obesity and cancer are multifactorial with significant contribution from genetics and environmental factors. *TP53* (p53) is the most commonly mutated gene in cancer with nearly half of all human cancers showing protein loss or mutation (2). Of the cancers that do not have mutations in the p53 gene locus, the majority exhibit mutations or altered levels of negative regulators of p53 (3, 4). Classically, p53 is known as a tumor suppressor, but recent work highlights the diverse functions of p53, including p53's contribution to metabolic and adipose tissue regulation. As increasing evidence links obesity to the onset of cancer, in this review, we discuss the crosstalk between adipose tissue and metabolism in cancer and the central role of p53 therein.

## p53 overview

**Figure 1:**
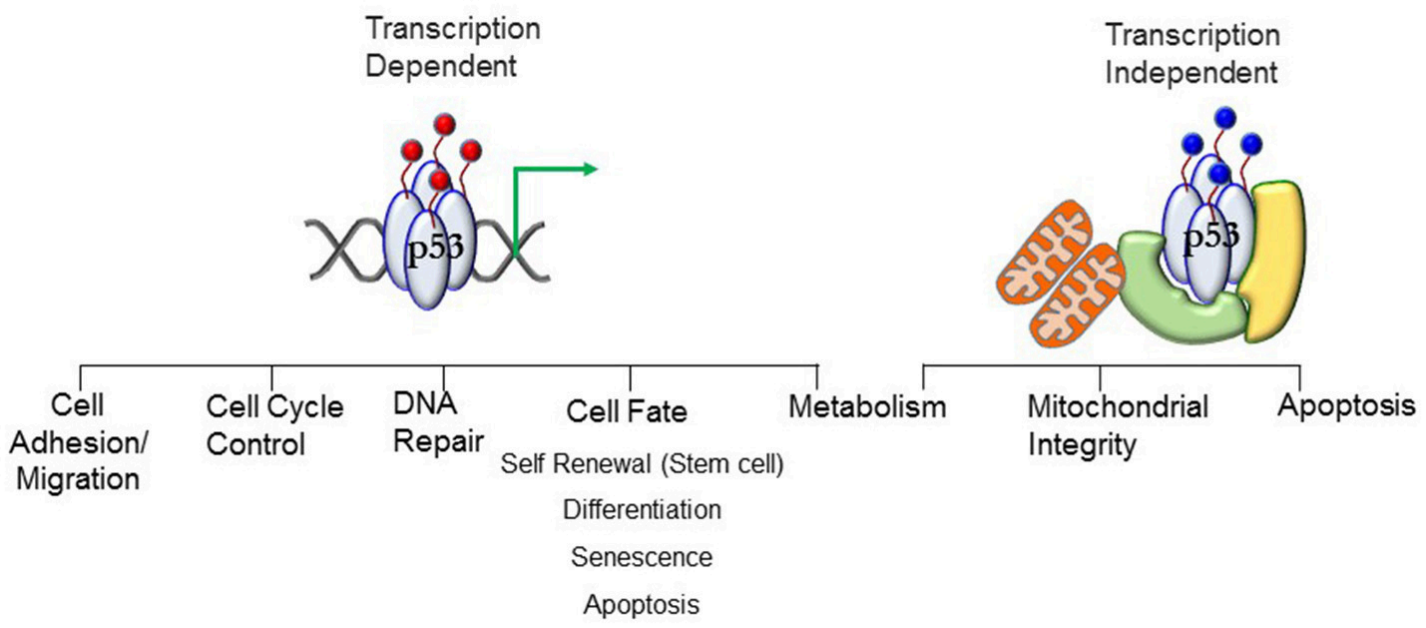
Transcript ion-dependent and -independent p53 function. Different combinations of posttranslational modifications (red circles)—the PTM signature—will dictate the context-specific transcriptional response that will translate to a phenotypic outcome. The PTMs dictate interactions of proteins with p53, enabling stimuli-specific cellular output. Transcription-independent response involving mitochondria require mono-ubiquitination (blue circles) of p53. Localization of p53 at the mitochondrial membrane and interaction with anti-apoptotic Bcl proteins stimulates apoptosis. Translocation into the mitochondrial matrix requires interactions with proteins (denoted in green and yellow) where it interacts with mitochondrial proteins to preserve mitochondrial integrity. See text for details.

p53 is best known as a tumor suppressor that maintains genomic stability and inhibits cell proliferation pathways (5–11). Its significant role in tumor suppression is dependent on its activity as a transcription factor regulating expression of genes in cell cycle regulation, apoptosis, DNA repair, differentiation, and senescence pathways (Figure 1). Under conditions of mild stress, p53 initiates cell cycle arrest and DNA repair pathways. However, in response to catastrophic stress that inflicts irreparable damage, p53 triggers an apoptotic response designed to limit propagation of impaired cells.

p53 protein levels are ubiquitously high in early embryogenesis in germ layer progenitors and embryonic stem cells until nearly mid-gestation (5, 12, 13), after which time expression is restricted to specific tissues during organogenesis as development progresses. Protein levels decrease postnatally to follow the recognized expression pattern of stabilization under cellular stress (5, 12, 14). Stimuli-induced post-translational modifications (PTM) stabilize the protein (15–21). In the absence of stress stimuli, negative regulation of p53 function is mediated by Mdm2 and Mdmx (22, 23). Different combinations of PTMs—the PTM signature—drive context-specific pathway activation. Protein stability and function are controlled by: (a) phosphorylation (b) acetylation (c) poly-ubiquitination (d) sumoylation (e) neddylation and (f) methylation (17, 21, 24–28). The *N*-terminus contains the transcription activation domain (TAD). In addition to stabilizing p53, the PTMs dictate the interactions of proteins with p53, enabling stimuli-specific cellular output. For example, p53 interacts with histone modifying enzymes and chromatin remodelers [e.g., HATs p300/CBP (29, 30), lysine-specific demethylase LSD1 (31)] which alter chromatin structure, along with interactions with proteins in the basal transcription machinery complex [TBP (32), and TBP-associated factors such as TFIIA and TAF1 (33, 34)] to regulate gene transcription (33, 35, 36). Transcription-dependent functions of p53 play a key role in cell-fate decisions by regulating expression of genes that control cell cycle arrest, DNA repair, apoptosis, senescence, and autophagy to limit the propagation of cells with damaged genomes (33–36).

Research in the last decade has revealed a critical role for p53 well beyond its role in tumor suppression. These roles include preserving stem cell health and differentiation in embryonic life, development of senescence and maintaining mitochondrial function in aging (5, 7, 37–41). Recent evidence strongly implicates p53 in the regulation of metabolism, linking p53 to metabolic abnormalities observed in aging, obesity, inflammation, and cancer (37, 42).

## p53-mediated regulation of intermediary metabolism

Choice of metabolic pathway usage is determined by the cell's energy, biomass and metabolite demands. Many cancer cells depend on glycolysis, even under aerobic conditions (Warburg Effect) (43, 44). The shift to aerobic glycolysis is an active reprogramming event that enables anabolic growth. Intermediates from the glycolytic pathway serve as precursors for biomass synthesis that are necessary for proliferation. Additionally, the pentose phosphate pathway (PPP) produces precursors for the synthesis of nucleotides that are essential for DNA replication. In contrast, differentiated cells preferentially utilize mitochondrial oxidative phosphorylation (OXPHOS) (45).

Consistent with its role as a tumor suppressor, p53 inhibits multiple steps of glycolysis and the PPP while promoting OXPHOS (46). Expression of glucose transporters Glut1 and Glut4 are downregulated by p53, resulting in the inhibition of glucose uptake. Induction of the phosphatase TP53-induced glycolysis and apoptosis regulator (TIGAR) decreases the production of fructose-2,6-bisphosphate (F2, 6BP) which allosterically activates phosphofructokinase 1 (PFK1) to increase glycolytic flux (9). By inhibiting expression of the negative regulator of the pyruvate dehydrogenase complex that is responsible for the transfer of cytosolic pyruvate to the mitochondria, p53 promotes OXPHOS by directing pyruvate to acetyl CoA rather than lactate (47). Increased lactate levels in the cell due to transcriptional repression of monocarboxylate transporter 1 (*mct1*) expression, a p53 target gene which transports lactate out of the cell, also decreases glycolytic flux (48).

p53 is a critical regulator of mitochondrial morphology, mitochondrial genomic integrity, mitophagy, aerobic metabolism and cellular redox state (38, 41, 49). In contrast to inhibitory effects on anabolic glycolysis, p53 drives catabolic mitochondrial respiration via induction of key genes such as mitochondrial glutaminase (*Gls2)*, Synthesis of cytochrome *c* oxidase 2 (*Sco2*) and Complex 1 proteins that are involved in fueling the tricarboxylic acid (TCA) cycle and driving electron transport (50, 51). p53 was demonstrated to adaptively regulate OXPHOS in Drosophila Myc+ cells and maintain their super-competitive status by enhancing the metabolic flux (52). In contrast to increased proliferation observed in cancer cells upon the loss of p53, the response of Drosophila Myc+ cells to p53 loss is impaired metabolism and reduced viability, suggesting a cell-context dependent regulation of cellular processes. By inducing expression of the mitochondria-eating protein (Mieap), p53 functions as a guardian of mitochondrial health, facilitating the removal of damaged mitochondria by mitophagy (53). Mitochondrial p53 physically interacts with TFAM, the factor that is responsible for mitochondrial DNA transcription, replication, and repair (11). Accordingly, decreased mitochondrial DNA content or mitochondrial DNA mutations are detected in fibroblasts from Li-Fraumeni patients (54).

p53 also plays a critical role in both normal and pathological lipid metabolism (55, 56). Generally, p53 is a negative regulator of lipid synthesis and activates fatty acid oxidation (FAO) via induction of expression of carnitine acetyltransferase genes (CPT1) that transport fatty acids to the mitochondria for oxidation. However, chronic p53 activation by nutrient stress (obesity) leads to hepatic steatosis, insulin resistance, and diabetes, pointing to the complexity of the homeostatic response (57–59). Dysregulated cell metabolism is an accepted hallmark of cancer and p53 can influence the function of many metabolic pathways (60). Obesity is also recognized as a state of dysregulated cell metabolism, and p53 is influential in adipose tissue differentiation, accumulation, and cytokine secretion.

## Adipose tissue

Adipose tissue is broadly subdivided into white and brown adipose tissue. The largest component of white adipose tissue is the large, spherical adipocyte with a unilocular lipid droplet occupying most of the cell volume. The primary role of white adipose tissue is to store energy in the form of triglycerides. When hormones signal the need for energy, fatty acids and glycerol are released through lipolysis. White adipose tissue is subdivided into unique depots highlighting the function and location of the adipose tissue. Visceral adipose tissue surrounds organs, subcutaneous adipose tissue forms a layer between the muscle and dermal fascia, and intramuscular adipose tissue protects tissue and supplies nourishment. Approximately 80% of human adipose tissue is deposited in subcutaneous depots. However, visceral adipose tissue is more metabolically active, and its accumulation is more prognostic of obesity-related mortality (61, 62). Both white adipose tissue depots store excess energy, but visceral fat also protects organs from physical trauma. White adipose tissue is capable of significant expansion that can lead to the accumulation of excess adipose tissue and thus increased propensity for obesity and related metabolic disorders (63).

In contrast to white adipose tissue, brown adipose tissue is specialized to burn sugars and lipids to generate heat and to help maintain body temperature through adaptive thermogenesis. Brown adipose tissue is abundant in neonates but undergoes rapid involution with age in humans. Consequently, adult human brown adipose tissue is relatively limited in mass and restricted to depots near the aorta and within the supraclavicular region of the neck (64). Brown adipose tissue is densely innervated by the sympathetic nervous system and is highly vascularized. Brown adipocytes contain multilocular lipid droplets and large numbers of mitochondria. The hallmark of brown adipose tissue function is the presence and activation of mitochondrial uncoupling protein 1 (UCP1) which uncouples OXPHOS from ATP synthesis in the inner mitochondrial membrane, thereby dissipating chemical energy as heat (65). A third adipose tissue type termed beige or “brown-in-white” (brite) adipose, has recently been characterized. Beige adipocytes can be induced by cold and a broad spectrum of pharmacological substances and, therefore, they are also known as “inducible brown adipocytes.” These depots can be induced to appear morphologically similar to brown adipose tissue, but appear in classical white adipose tissue depots and are derived from a non-classical brown adipose tissue lineage (66, 67).

Recently, the bone marrow has been identified as a unique adipose depot. Although the bone marrow contains few adipocytes at birth, the number increases with age, and by adulthood, bone marrow adipose tissue constitutes over 10% of the total fat mass in lean, healthy humans. There are two types of bone marrow adipose tissue classified as “regulated” that may influence hematopoiesis and “constitutive” that is important during early vertebrate development (68). The ontogeny of bone marrow adipose tissue is not well defined. Bone marrow adipose tissue differs in diet response, phenotype, gene expression and physiological actions from other adipose depots [reviewed in (69)]. For example, during conditions of starvation bone marrow adipose tissue volume increases whereas white adipose tissue volume decreases.

It is now clear that all adipose tissue acts in an autocrine/paracrine and endocrine manner. Adipocytes secrete an array of signaling molecules such as leptin, adiponectin, plasminogen activator inhibitor (PAI-1), vascular endothelial growth factor (VEGF), tumor necrosis factor-alpha (TNF-α), and interleukin (IL)-6, collectively referred to as adipokines, that communicate with other organs such as the brain, liver, muscle, the immune system, and adipose tissue itself. An example is metabolic symbiosis that occurs between tumor cells and adjacent adipose tissue during cancer progression. Adipokines and lipids are released from mature adipocytes and taken up by cancer cells. Paracrine factors from adipose tissue-derived stromal and immune cells that have infiltrated tumors, are secreted into the tumor microenvironment (70).

Differentiation of preadipocytes to mature adipocytes requires transcription regulators such as the peroxisome proliferator-activated receptor gamma (PPARγ) and members of the CCAAT/enhancer-binding protein family (C/EBPs) (71). p53 is a negative regulator of PPARγ expression, and concomitantly of white adipocyte differentiation both *in vivo* and *in vitro* (72). p53 inhibits an adipogenic program in 3T3-L1 preadipocytes and mouse embryonic fibroblasts (MEFs) (73, 74). Knockdown of p53 by specific shRNA enhances the adipogenic capacity in both mouse and human cell lines, indicated by increased levels of adipogenic markers such as PPARγ, AP2, and adiponectin even without hormonal induction (74). Moreover, differentiation of p53-null MEFs into adipocytes is more robust compared to wild-type cells in an adipogenic medium (73–75). Accordingly, transgenic mice overexpressing active p53 demonstrate decreased adipose tissue deposition and reduction in body mass (76). However, p53 is a positive regulator of brown adipocyte differentiation (75). Also, using a murine model of diet-induced obesity (DIO) weight gain was reduced in p53-null mice, and the mechanism was through an increase in UCP1 expression, both in brown and white adipose tissue (77).

## Adipose tissue dysfunction—promoted by p53?

**Figure 2:**
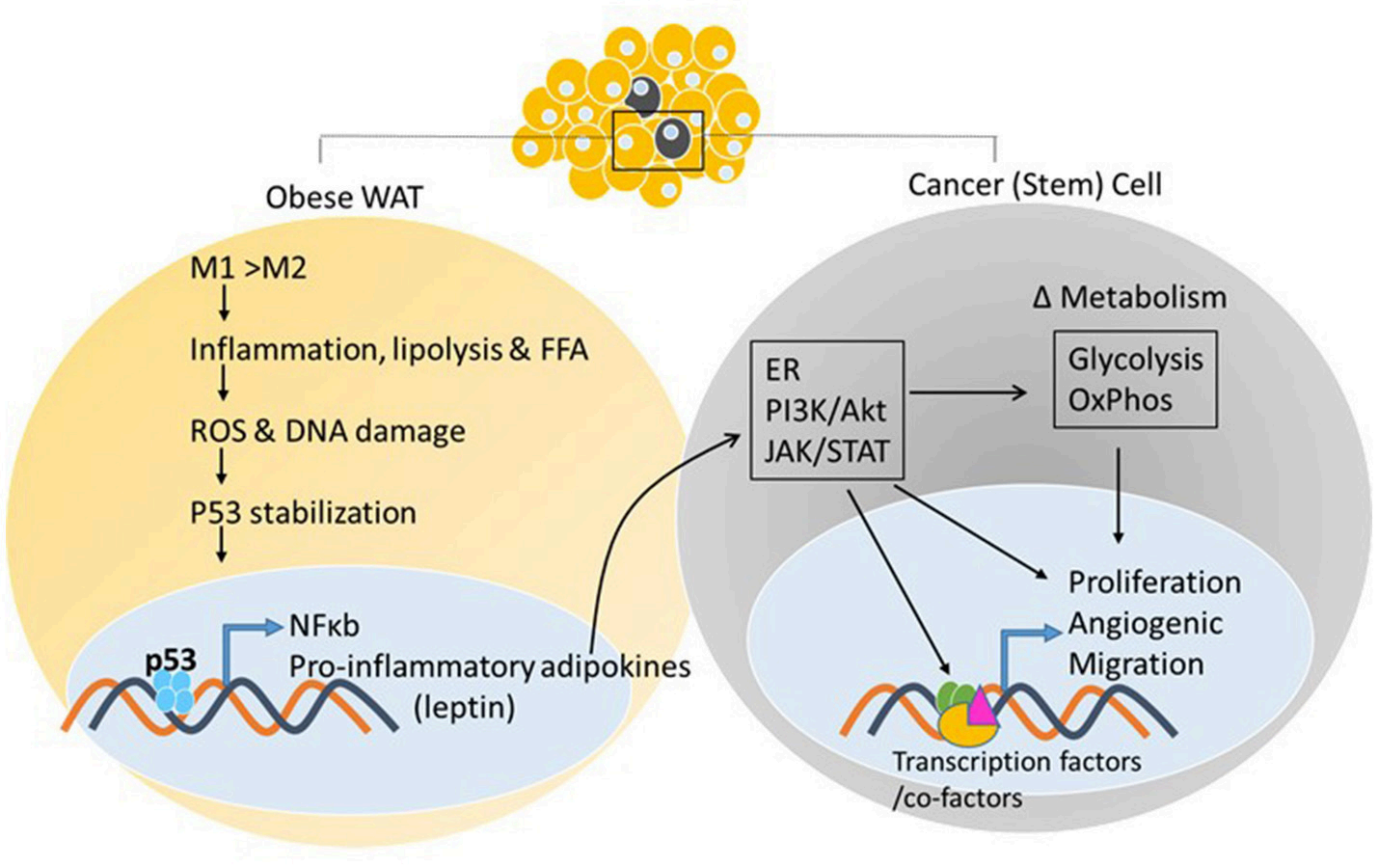
White adipose tissue and cancer. Excess adiposity increases the proportion of proinflammatory M1 to anti-inflammatory M2 macrophages in white adipose tissue, resulting in production of inflammatory cytokines and stabilization of p53, further increasing adipokine transcription via p53/NF-κb activation. Adipokine release from the white adipose tissue and action on the neighboring tumor cell, or cancer stem cell, activates oncogenic signaling pathways that can impact energy metabolism pathways and transcriptional output via post-translational modifications of enzymes, transcription factors/co-factors, respectively, facilitating tumor initiation/promotion.

**Figure 3:**
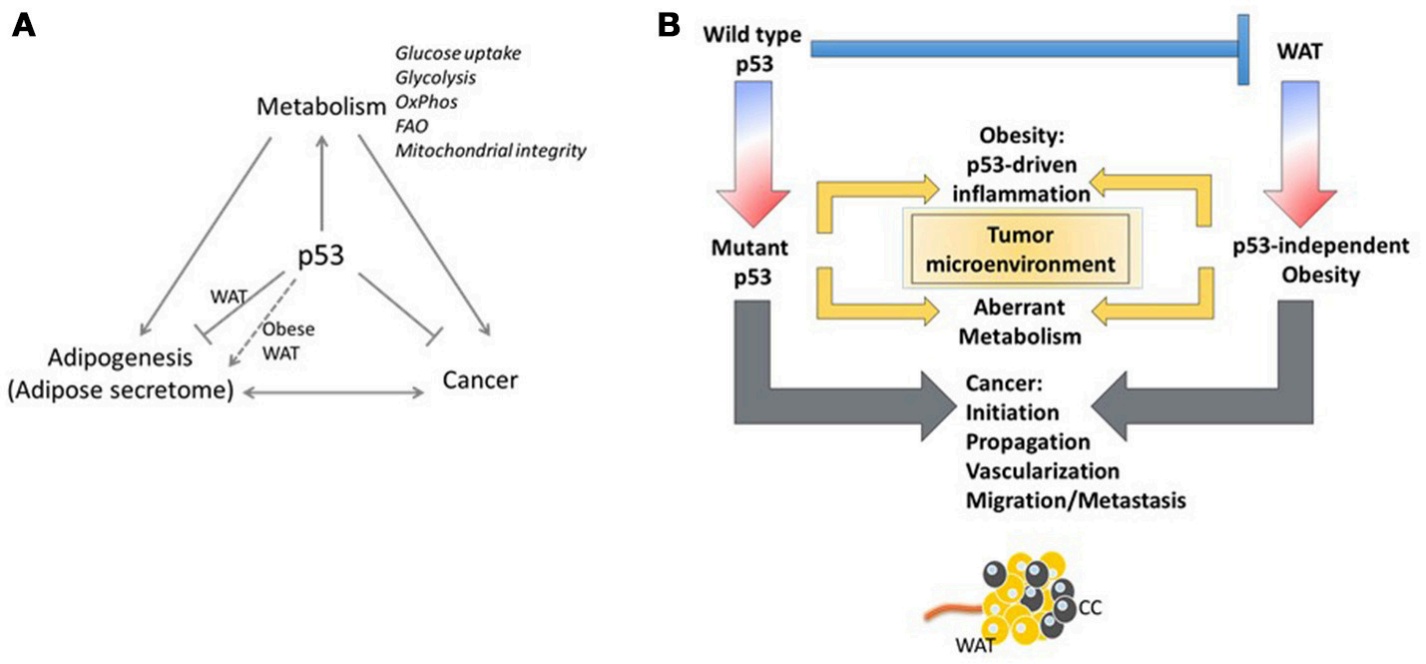
Yin and Yang of p53. **(A)** The p53/white adipose tissue/Cancer nexus. **(B)** The adipose tissue microenvironment contributes significantly to malignancy through tumor microenvironment communication. Regardless of the p53 status of the tumor cell, stimulation of oncogenic signaling pathways by p53-dependent adipokine production from the white adipose tissue in the microenvironment may facilitate tumor propagation.

As adipose tissue expands, adipogenesis is upregulated, mature adipocytes enlarge, and angiogenic processes promote neovascularization. In obese states, enlarged adipocytes experience hypoxic conditions due to larger distances from the vasculature (78), as cardiac output and total blood flow do not increase with increased obesity (79). In association with these changes, the adipose tissue starts to produce chemotactic factors, such as monocyte chemoattractant protein (MCP)-1, that attract monocytes/macrophages into adipose tissue (80). Murine studies have demonstrated that excess adiposity increases the proportion of proinflammatory M1 to anti-inflammatory M2 macrophages in white adipose tissue (81). As the adipose tissue becomes inflamed, production of inflammatory cytokines increases and production of adiponectin decreases, resulting in the inability to store surplus free fatty acids (FFAs) leading to further adipose tissue dysfunction (82). *In vitro* and *in vivo* studies by Shimizu et al. indicated that increased release of FFAs led to ROS-induced DNA damage and upregulation of p53 in adipose tissue (59) (Figure 2). Activation of p53 upregulated the expression of proinflammatory adipokines via the NF-κB signaling pathway, and promoted adipose tissue inflammation, insulin resistance, and diabetes, whereas inhibiting p53 activity attenuated the inflammation (59). These changes in p53 expression related to obesity have been observed in both murine models and obese human subjects (55, 58, 83–86). The chronic inflammation associated with dysfunctional adipose tissue is thought to contribute to a favorable microenvironment for tumor growth and progression (Figures 2, 3).

In addition to data indicating p53 stimulation in dysfunctional adipose tissue exacerbates the pathology of adiposity, recent studies implicate p53 as a primary mediator of adiposity. As demonstrated by Kung et al. mice harboring the proline-to-arginine 72 (P72R) variant of p53 developed more severe obesity and glucose intolerance on a high-fat diet than mice with proline 72 variant (87). Further evidence supporting the adverse effect of high p53 activity in promoting obesity was demonstrated in mutant MDM2^C305F^ mice that have impaired p53 regulation of lipid metabolism (88). The mutation disrupts ribosomal protein-MDM2 interaction that serves to sequester MDM2 and allow p53 activation. Also, pharmacological inhibition of p53 was demonstrated to prevent high-fat diet-induced weight gain observed in control mice (89). In summary, the data suggest that high p53 levels, whether induced in response to or as an inducer of adiposity, are likely counter-productive in maintaining adipose tissue homeostasis.

## p53, adipose tissue, and metabolism—an unexplored link in cancer

Secretion of adipokines (leptin, adiponectin, endotrophin, etc.) and growth factors from AT promote tumor growth. There are more than 600 different adipokines currently identified and many cancers, such as breast cancer, have adipokine receptors present on the cancer cells (90, 91). Adipokine-linked cancer progression may occur through increased proliferation, migration, inflammation and anti-apoptotic mechanisms. Leptin secretion from adipose tissue near tumors is increased, but not in adipose depots that are distant from the tumors (92). Interestingly, leptin is a known regulator of p53 expression (93). Leptin binding to its receptor enhances the proliferation and growth of breast cancer cells through numerous signaling pathways including estrogen receptor, JAK/STAT3, and PI3K/Akt pathways (94–96) (Figure 2). Aberrant signaling through these pathways activates expression of genes that contribute to cancer cell survival, proliferation, and migration (97–99). Moreover, signaling pathway activation can reprogram cellular metabolism to support the specific metabolite demands of proliferating cells. Thus, ectopic activation of these pathways promotes tumor progression (Figure 2). Leptin was also shown to induce aromatase and this correlated positively with BMI, leading to increased risk for breast cancer (100) (Table 1). Given the participation of p53 in adipose tissue inflammation (as discussed above in section Adipose Tissue Dysfunction—Promoted by p53?) that promote proliferative pathways vs. the known involvement of p53 as a tumor suppressor restricting proliferation and cell growth, the role of p53 in adipose tissue-driven tumorigenesis remains to be elaborated.

**Table 1:** p53 and adipose tissue metabolism.

**Type of cancer**	**Model**	**Mechanism**	**Cancer-related outcome**	**References**
Breast cancer	Primary breast adipose stromal cells	Prostaglandin E2 (PGE2) decreases p53 expression and increases aromatase levels.	Increased aromatase is associated with increased estrogen production	(101)
Breast cancer	Primary preadipocytes	Leptin-mediated induction of aromatase was dependent on PKC/MAPK signaling and inhibition of p53	Increased aromatase is associated with increased estrogen production	(100)
Hepatocellular cancer	HepG2 and HuH-7 cell line	Omentin-1 upregulated p53 through sirtuin1-dependent deacetylation of p53	Apoptosis	(102)

Wild-type p53 in an inactivated or dysfunctional form accumulates in the cytoplasm whereas stable p53 binds to target genes in the nucleus. Expression of the p53 transcript, nuclear localization of the protein and phosphorylation at Ser15 was decreased in ASCs due to the effect of prostaglandins (PGE_2_) (101). Wang et al. showed that the decrease in p53 protein expression and activity is through an inhibitory effect of PGE_2_ on AMP-activated kinase (AMPK). AMPK can no longer phosphorylate p53 at Ser15 (103, 104), resulting in decreased nuclear localization and transcriptional activity of p53. In clinical samples of breast cancer, tumor-associated ASCs had reduced nuclear p53 staining and increased perinuclear staining compared to normal ASCs (101). This is important as increased PGE_2_ is linked with many cancers and PGE_2_ associated inflammation is specifically associated with obesity and breast cancer (105). PGE_2_ and TNFα may contribute to the Warburg effect due to stimulation of GLUT1 and GLUT3 in ASCs (106). Again, this mechanism is through adipose-derived inflammation altering the metabolic microenvironment resulting in reduced p53 nuclear localization. A mechanism to the observed obesity-associated increase in aromatase and its link to breast cancer has been suggested (100). Adipose or ASC leptin secretion resulted in activation of PKC/MAPK signaling pathways and inhibition of p53. Furthermore, HIF1α and PKM2 were stabilized, resulting in increased expression of aromatase, and an increased risk of estrogen-dependent breast cancer. Conversely, p53 related mechanisms have been shown to promote hepatocellular carcinoma cell apoptosis. Omentin-1, an adipokine, was added to hepatocellular carcinoma cells and resulted in an inhibition of proliferation and an induction of apoptosis (102). It was shown that omentin-1 upregulated p53 through sirtuin1-dependent deacetylation of p53. This is in contrast to the actions of most reports on adipokines and cancer, which show promotion of metastatic potential and cancer cell survival.

Obesity has long been linked to increased local inflammation. As discussed above, obesity also reprograms metabolism systemically and can lead to increased levels of glucose and dyslipidemia in the blood (107). Although these examples are associated with obesity, the distribution of adipose tissue results in proximate or direct contact of tumors with adipose tissue, both in obese and non-obese conditions. This growing field of study suggests that the adipose tissue microenvironment contributes significantly to malignancy through tumor-microenvironment communication (Figure 3). In an invasive ductal carcinoma breast cancer model, increased lymph node metastasis was reportedly linked to adipose tissue invasion at the tumor margin (108). Tumor cells have been reported to induce delipidation of adipocytes and promote lipolysis in the tumor microenvironment (109). Regardless of the p53 status of the tumor cell, stimulation of oncogenic signaling pathways by p53-dependent adipokine production from the white adipose tissue in the microenvironment may facilitate tumor propagation (Figure 3).

Finally, bone marrow adipose tissue (BMAT) has recently been shown to affect metastatic progression and drug resistance in prostate and breast cancer. The mechanisms involved in this new adipose depot are currently being resolved. Fairfield et al. used a 3D culture model of BMAT to show that BMAT adipocytes, when co-cultured with tumor cells, undergo delipidation (110). This supports the model of exogenous lipid dependency by tumor cells for metabolic flexibility within the metastatic niche. Lipids from adipocytes in the tumor microenvironment could potentially regulate metabolic and signaling pathways in cancer cells, providing them with a survival advantage. A role for p53 in bone marrow adipose tissue has not yet been investigated.

## Conclusions and future directions

An increased risk of cancer development and a poorer cancer prognosis is associated with increased obesity (107, 111–114). Cancer survivors with a higher body mass index are more likely to experience a cancer recurrence (115). The mechanisms linking increased adiposity to malignancy are not entirely understood. Altered interactions between adipose tissue and systemic or neighboring tissue, changing endocrine hormone and adipokine secretion that would facilitate tumor invasion and metastasis are hypothesized to drive metabolic reprogramming in tumor cells and provide metabolites and lipids required for tumor progression and growth. Although brown adipose tissue is metabolically more active than white adipose tissue, the link between chronic metabolic diseases and brown adipose tissue is unknown. Given the differential regulation by p53 of white vs. brown adipose tissue, it will be interesting to compare the influence of these different adipose depots for their potential to contribute to cancer. A thorough understanding of the crosstalk between cancer cells and the adipose microenvironment may well reveal novel therapeutic targets for cancer treatment.

## Author contributions

All authors listed have made a substantial, direct and intellectual contribution to the work, and approved it for publication.

### Conflict of interest statement

The authors declare that the research was conducted in the absence of any commercial or financial relationships that could be construed as a potential conflict of interest.
